# P110 A complex case of juvenile-onset diffuse systemic sclerosis: biopsychosocial and transitional care challenges

**DOI:** 10.1093/rap/rkag113.140

**Published:** 2026-11-03

**Authors:** Ryan Lethem, Frances Hall, Peter Bale

**Affiliations:** Cambridge University Hospitals NHS Foundation Trust, Cambridge, United Kingdom; Cambridge University Hospitals NHS Foundation Trust, Cambridge, United Kingdom; Cambridge University Hospitals NHS Foundation Trust, Cambridge, United Kingdom

## Abstract

**Introduction:**

We describe the case of a 16-year-old female with a new diagnosis of diffuse systemic sclerosis. Her phenotype included significant skin thickening, dysphagia and interstitial lung disease. Her diagnostic journey was tortuous, predominated by unexplained weight loss and pruritis, alongside a challenging psychosocial background.

The rare, ambiguous, yet potentially severe disease course of juvenile diffuse systemic sclerosis makes this case in itself worthy of discussion. However, it also highlights the challenges of identifying the best location for developmentally appropriate care in young people aged 16-17 years, particularly those with a suspected severe chronic condition requiring urgent management.

**Case description:**

A 16-year-old female presented with a twelve-month history of progressive unexplained weight loss. Early symptoms included pruritis and Raynaud’s phenomenon, progressing to skin tightness, painful digital ulcers, myalgia and dysphagia. An autoantibody screen revealed a positive antinuclear antibody (ANA) and anti-Scl70 antibody.

Due to her age, she was initially referred by general paediatrics to adult rheumatology and was suspected to have diffuse systemic sclerosis. She was not commenced on treatment and instead was unsuccessfully referred to specific adolescent rheumatology services. A review by visiting paediatric rheumatology initiated a plan to electively admit her under paediatrics, facilitating joint adult rheumatology team management in a tertiary centre.

On assessment, she had diffuse skin thickening (Modified Rodnan Skin Score 24/51). There were painful ulcerations on her digits and elbows. Nailfold capillaroscopy showed active and atrophic changes. There was no evidence of arthritis, muscle weakness or respiratory abnormalities.

Investigative work-up included a hr-CT thorax, which showed basal interstitial changes and an oesophageal fluid level, raising the suspicion of oesophageal dysmotility. She reported some dysphagia and her swallow safety was assessed. Despite no overt respiratory symptoms, a pulmonary function test was suggestive of impaired gas exchange and forced vital capacity. An echocardiogram demonstrated no pulmonary hypertension and a CT abdomen-pelvis showed no visceral inflammation. Her blood and urine tests were largely unremarkable, suggesting no other organ involvement.

She was commenced on oral prednisolone (1mg/kg OD weaning over 6 weeks) and mycophenolate mofetil (initially 500mg (360mg/m^2^) BD), lansoprazole 30mg OD, and sildenafil 25mg BD.

She will have interval pulmonary function tests after establishing treatment and will receive a barium swallow to further elucidate her oesophageal symptoms. She will remain under joint care of paediatric and adult rheumatology services until she is established on a stable treatment regimen and after the safe transition process can be completed.

**Discussion:**

Systemic sclerosis is a rare, complex connective tissue disease, characterised by immune dysregulation, vasculopathy and excess collagen deposition, leading to progressive organ fibrosis. It has an incidence of 2.9 per million person-years in children aged <16 years and a prevalence of 3 in 1,000,000 in those aged 0-17 years.

Multisystem involvement can lead to significant morbidity, ranging from functional disability from sclerodactyly and calcinosis, to severe organ dysfunction, including interstitial lung disease, pulmonary hypertension and renal crisis, and an increased mortality rate.

This case highlights major learning points. Firstly, managing her multisystem disease required targeted treatment. Mycophenolate Mofetil was chosen as first line immunosuppression primarily for skin and lung involvement, per BSR 2024 guidance.

Prednisolone was used as an adjunct. The 2024 guidance does not recommend restricting glucocorticoids in children, as the risk of renal crisis is significantly less compared to adults, and manages overlap symptoms more commonly seen in children. Curiously, as this patient was co-managed by both adult and paediatric teams, there was conflicting opinion on commencing glucocorticoids.

Secondly, this case highlights the wider challenges encountered by patients presenting to rheumatology for the first time aged 16-17 years. Local care arrangements in this population often expedite a referral to adult teams from the outset. However, this case highlighted the potential pitfalls of this process. In addition to the previously mentioned treatment delays, this patient had wider psychological needs and had a complex social background; further, the services of the wider multidisciplinary team are often more available in paediatrics compared to adult services, highlighting the wider issue of this population defined legally as a child, but whom are often processed through adult services.

We hope highlighting this case leads to increased awareness of this condition, and may stimulate ideas for addressing the challenges faced in the 16-17 year-old population.

**Key learning points:**

• Systemic sclerosis is a rare and complex condition with high rates of morbidity and mortality. Initial symptoms may be ambiguous and insidious; early recognition can help mitigate poorer outcomes.

• Once diagnosis is established, broad investigative workup includes assessment of the skin, lungs, kidneys, heart and upper GI system.

• Treatment is variable depending on the extent and type of organ involvement. MMF is typically the first line systemic treatment. Glucocorticoids may be used more readily in children compared to adults.

• Local transitional services for children diagnosed aged 16-17 years may face additional policy and logistical challenges in accessing care quickly.

• Addressing the wider biopsychosocial needs of those in the 16-17 age bracket transitioned directly to adult services may be more challenging, as there is often a reduced access to wider multidisciplinary team services compared to paediatrics.

**Learning Objectives**

1. To highlight a rare case of systemic sclerosis with juvenile onset, detailing the phenotype, diagnostic work-up and management

2. To highlight the additional challenges of local and transitional care policies where the patient presents aged 16-17 years. We would encourage discussion from delegates from different centres to share their own local practices, and to identify possible ideas for improvement strategies in the delivery of care to such patients

